# Potential Anti-HPV and Related Cancer Agents from Marine Resources: An Overview

**DOI:** 10.3390/md12042019

**Published:** 2014-04-03

**Authors:** Shi-Xin Wang, Xiao-Shuang Zhang, Hua-Shi Guan, Wei Wang

**Affiliations:** 1 Key Laboratory of Marine Drugs, Ministry of Education, Ocean University of China, Qingdao 266003, China; E-Mails: shixinwang87@gmail.com (S.-X.W.); xiaoshuangzhang89@126.com (X.-S.Z.); hsguan@ouc.edu.cn (H.-S.G.); 2 Shandong Provincial Key Laboratory of Glycoscience and Glycoengineering, Ocean University of China, Qingdao 266003, China

**Keywords:** human papillomavirus, cervical cancer, pathogenesis, heparinoid polysaccharide, entry process, apoptosis

## Abstract

Recently, the studies on the prevention and treatment of human papillomavirus (HPV) which is closely related to the cervical cancer and other genital diseases are attracting more and more attention all over the world. Marine-derived polysaccharides and other bioactive compounds have been shown to possess a variety of anti-HPV and related cancer activities. This paper will review the recent progress in research on the potential anti-HPV and related cancer agents from marine resources. In particular, it will provide an update on the anti-HPV actions of heparinoid polysaccharides and bioactive compounds present in marine organisms, as well as the therapeutic vaccines relating to marine organisms. In addition, the possible mechanisms of anti-HPV actions of marine bioactive compounds and their potential for therapeutic application will also be summarized in detail.

## 1. Introduction

Papillomavirus is a diverse group of non enveloped DNA viruses that infect the skin and mucosal tissues of a range of vertebrate species, including humans [1,2,3]. In recent years, human papillomavirus (HPV) was confirmed existing in reproductive tract and other organs, including respiratory, bladder, mouth and throat, and its pathogenesis was dependent on virus serotype, the site of infection, and the immunity [4,5]. Infection with genital HPV types is very common, with an estimated lifetime risk of infection of about 75% [6]. Although most genital HPV infections are subclinical and self-limiting, a subset of persistently infected individuals has lesions that progress to premalignancy or cancer [7]. Virtually 100% of cervical, ~43% of vulvar, and ~70% of vaginal tumors are attributable to human papillomavirus infection, which annually generating 530,000 cervical and 21,000 vulvar and vaginal cancers worldwide [6,8].

Although HPV infection is considered a sexually transmitted infection, HPVs can also be transmitted by on-sexual routes including casual physical contact and erinatal vertical transmission [9,10]. Some studies have suggested that condoms are, at best, only marginally effective for preventing the sexual transmission of HPV [11,12]. Recently, a highly effective group of prophylactic HPV vaccines are becoming publicly available in the developed countries [13]. However, there are two possible drawbacks for these vaccines that they are relatively expensive (at least initially) and are likely to be papillomavirus type-restricted in their protection [7]. Thus, the vaccines may not initially be available to women in all parts of the world and may not offer protection against all cancer associated HPV types. Therefore, the search for potential drug candidates with higher inhibitory activities against various HPV strains is increasing in the pharmaceutical industry nowadays. In this regard, marine derived natural bioactive compounds and their derivatives are great sources for the development of new generation anti-HPV therapeutics, which is more effective with fewer side effects.

This review presents an overview of recent progress in research on the prophylaxis and therapy for HPV and related cancer diseases. Moreover, this review will mainly focus on the heparinoid polysaccharides and bioactive compounds present in marine organisms. Recent developments in the possible mechanisms of anti-HPV actions of marine bioactive compounds and their potential for therapeutic application will also be discussed in detail.

## 2. Update on Pathogenesis and Therapy for HPV and its Related Cancer

### 2.1. The Pathogenesis of HPV and Its Related Cancer

Human papillomavirus (HPV) is a diverse group of non-enveloped DNA virus appearing icosahedra symmetry spherical particles with diameters of ~45 to 55 nm, and contains more than 130 serotypes [2,3]. HPV genome is a ~8 kb circular DNA, which codes for about 6 early (*E1*, *E2*, *E4*, *E5*, *E6* and *E7*) and two late genes (*L1* and *L2*) [14,15,16]. On the basis of their association with disease types, papillomaviruses are classified into high-risk (HR) and low-risk (LR) types [14]. HR-HPV types (HPV 16, 18, 31, 33, 35, 39, 45, 51, 52, 56, 58, 59, 66, 68, 70) are often associated with high grade lesions and invasive cancer, whereas the LR-HPV types (HPV 6, 11, 12, 13, 15, 32, 34, 40, 42, 43, 44, 53, 54) are mainly found in low grade lesions, genital or skin warts and condyloma accuminata [14,17,18].

HPV infects squamous epithelial cells in the cervix, glans of the penis, penile shaft, scrotum and anal verge by interacting with putative host cell surface receptors such as heparan sulfate proteoglycans [19] and alpha 6 integrins [20]. The virus enters the host cell via clathrin-mediated or caveolin-mediated endocytosis, depending on the HPV type [21,22]. The viral entry is the first step in HPV pathogenesis followed by its persistence in epithelial cells. These two steps establish the viral genome into the host cell and have been the subjects of therapeutic interventions by vaccines [14]. The third step of HPV life cycle is its integration into the human genome, which is associated with higher lesions. The fourth key step of HPV life cycle is expression of viral oncogenes in differentiating epithelium. All these four steps provide means of interference with HPV life cycle and thus represent targets for the development of anti-HPV drugs [14,23].

Moreover, HPV DNA integration is closely tied to the development of cancer, as most cases of HPV-induced cervical cancer feature an integrated form of the HPV genome [3,4]. In the carcinogenic process of HPV, *E2* gene normally integrates into the host cells, and result in the inactivation of E2 protein, which caused the over-expression of *E6*, *E7* genes [24]. Meanwhile, *E5* gene stimulates cell cycles via EGFR, which in turn raising the over-expression of *E6*, *E7* genes [25]. The activities of *p53* gene and *pRb* gene are inhibited by HPV E6 and E7 proteins, respectively [26,27], resulting in the activation of the transcription of human telomerase reverse transcriptase (hTERT) [28], and the imbalance of host cells, which eventually change the cells from the normal to the cancer. In summary, the carcinogenic process of HPV is a multi-factor and multi-step process, in which viral and host factors all play important roles.

### 2.2. HPV Vaccines

The viral entry is the first step in HPV pathogenesis, so some HPV vaccines targeting virus surface protein L1 can be used to prevention of HPV infection. There are two types of HPV vaccines including prophylactic and therapeutic vaccine [29,30,31,32,33], and recent studies show that virus like particles (VLP) could be the applied for preventive vaccine [13,34,35]. Vaccination with virus-like particles (VLP) has demonstrated efficacy in HPV prophylaxis but these vaccines lack therapeutic potential. Considering the vital role of HPV16 in carcinogenesis [3,36], most of the efforts for developing therapeutic HPV vaccines have been directed towards development of vaccines against HPV16 viral oncogenes *E6* and *E7* [37,38,39,40,41,42,43,44,45,46,47]. There are several groups of therapeutic vaccines including virus/bacterial vector vaccine, peptide antigens, recombinant protein vaccines and plasmid DNA vaccines [48]. The antigens of most therapeutic vaccines are the whole protein or peptides derived from HPV E6 and E7 proteins because of their oncogenic potential and they are invariably retained and expressed throughout HPV related disease progression and carcinogenesis [14]. Apart from therapeutic HPV vaccines, there have been attempts to pulse dendritic cells (DC) with tumor lysate expressing HPV16 antigens [49], which is currently being tested in clinic [43].

Moreover, it was reported that marine polysaccharide carrageenan was able to generate antigen-specific immune responses and anti-tumor effects in female (C57BL/6) mice vaccinated with HPV16 E7 peptide vaccine [50]. Furthermore, the enhancement was not restricted to E7 antigen but also applicable to other antigenic systems. Some other structurally similar compounds to carrageenan, such as dextran, can also generate similar immune enhancement [50]. Thus, carrageenan and its structurally related compounds may serve as adjuvants for enhancing peptide-based HPV vaccine potency. In summary, some marine derived polysaccharides could be used as adjutants to enhance the anti-HPV effects of HPV therapeutic vaccines.

### 2.3. Current Anti-HPV and Related Cancer Drugs

Even after establishment of causal relationship between HPV and cervical cancer, currently, presence or absence of HPV does not have any impact on deciding the treatment; the strategies are primarily anti-cancer rather than anti-viral [14], and there is still no FDA approved anti-HPV drug listed so far. Though scissor excision is the most preferred methods for genital warts, topical preparations of cytotoxic compounds like Podophyllin or Trichloroacetic acid are also utilized in USA and Europe [51]. The current anti-HPV drugs are mainly oral hormonal medicine such as acyclovir, ganciclovir, interferon and interleukin. Interferons (IFNs) are the only antiviral drugs approved for the therapy of benign HPV related lesions. However, the IFNα treatment has limited efficacy and not recommended for routine clinical practice in the treatment of high-grade HPV associated lesions [52].

Moreover, some traditional Chinese medicines possess good anti-HPV activities and have been used for prevention and treatment of HPV related cancer in China. Chinese medicine *chaihu* was reported to have good inhibition effects on HPV infection by interfering with the expression of HPV-DNA in genital warts [53]. Chinese medicine *Youdujing* can reverse the function of cervical lesions in high-risk HPV infected patients by inhibiting the expression of HPV-DNA [54]. Moreover, Chinese medicine *Paiteling* composed of *folium*, *sophora*, *cnidium*, *gall*, and *javanica oil* can eliminate or inhibit high risk HPV infection by destroying mitochondria and other membrane system selectively and then lead to cell degeneration and necrosis [55]. In addition, it was found that *Xinfuning* (recombinant human interferon α-2b vaginal effervescent capsule) could be applied to cure HPV infection in vagina by boosting NK cell activity, and *Xinfuning* combined with *Baofukang* suppository for treatment of patients with high-risk HPV infection was also effective [56].

However, these current anti-HPV drugs are usually expensive, easily lead to liver and kidney damage, and produce drug resistance after prolonged treatment. Therefore, the development of novel anti-HPV agents with low toxicity and high efficiency is of high importance.

## 3. Potential Anti-HPV and Related Cancer Agents from Marine Resources

### 3.1. Heparin and Marine Heparinoid Polysaccharides

Heparin is a member of the glycosaminoglycan (GAG) family of carbohydrates and consists of a variably sulfated repeating disaccharide unit. HPV have more than 100 kinds of serotypes, of which the type 5, type 11 [57,58] and the high risk sexually transmitted serotypes 16, 31, 33 and 39 [57,59] have been reported to be able to use heparin sulfate as a low affinity co-receptor on the cell surface. For example, HPV16 virus, which can cause cervical cancer, can bind to heparin sulfate through viral capsid protein L1. Moreover, some reports indicated that HPV-16/33 VLP could not infect the cell after heparanase acted on the COS-7 or HaCaT cell, and confirmed heparin sulfate is necessary for infection of HPV to cells [59,60,61,62]. Several heparan sulfate proteoglycans (HSPGs) can serve as HPV receptors and support a putative role for syndecan-1, rather than α6 integrin, as a primary receptor protein in natural HPV infection of keratinocytes [63]. A recent study showed that L1 binding to heparan sulfate makes the L2 protein and cyclophilin B together to promote the cyclophilin B-mediated conformational change of L2 protein, so that HPV can effectively invade host cells [64]. Furthermore, some studies have shown that the structure of heparan sulfate may also affect tissue tropism of HPV and other heparin sulfate-binding pathogens [57].

**Figure 1:**
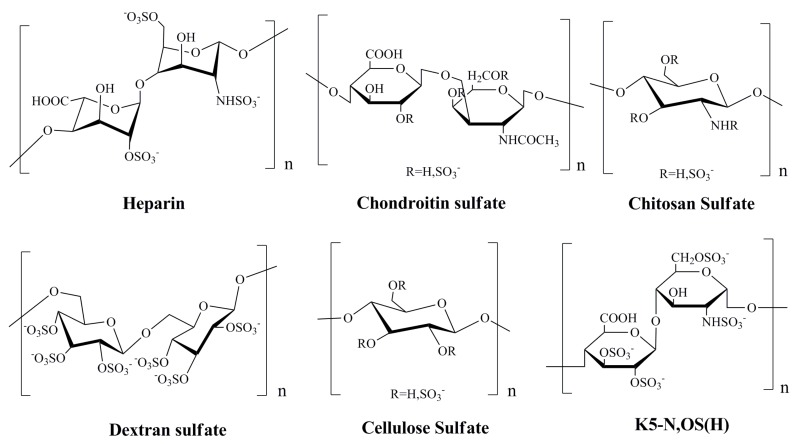
Chemical structures of the repeat units of heparin and heparinoid polysaccharides with anti-Human papilloma virus (HPV) effects [65,66,68].

The marine heparinoid polysaccharides are similar to heparin in structure, and possess GAG-like biological properties, which contain alginates, ulvans, and their sulfated derivatives, as well as the dextran sulfate and chitosan sulfate (Figure 1) [65]. It was reported that sulfated polysaccharides, such as heparin, cellulose sulfate and dextran sulfate, can block the infectivity of papillomaviruses [22,66]. For many classes of virus, including papillomaviruses, initial attachment of the virion to host cells is thought to be mediated mainly by interactions between the virion and a type of cell surface glycosaminoglycan known as heparan sulfate [67]. Many previous studies have indicated that some marine heparinoid polysaccharides such as alginic acid and fucoidan could effectively block HPV pseudovirion infection just like heparin [7]. Moreover, it was reported that the sulfated derivatives of Escherichia coli K5 capsular polysaccharides such as K5-N,OS(H), K5-N,OS(L), and K5-OS(H) which have a backbone structure resembling the heparin/heparan biosynthetic precursor can significantly inhibit HPV-16, HPV-18, and HPV-6 pseudovirion infection (IC_50_ < 1.0 µg/mL) [68].

In summary, the sulfate polysaccharides such as heparin and heparinoid polysaccharides can inhibit the entry process of HPV by interfering with the initial attachment of the viral particle to the host cell, which suggests these polysaccharides can be developed into novel anti-HPV drugs in the future.

### 3.2. Polysaccharides Derived from Red Algae

**Figure 2:**
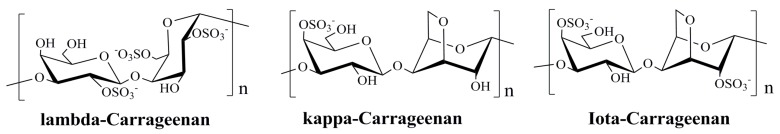
Average structures of the repeat units of λ-, κ- and ι-carrageenans [76].

Carrageenan, a sulfated polysaccharides of d-galactose and 3,6-anhydro-d-galactose extracted from red algae (Figure 2), has been widely used in use as a thickener in a variety of cosmetic and food products, ranging from sexual lubricants to infant feeding formulas [7]. Many researches indicated that carrageenans possess good inhibitory effects on different viruses such as HIV, HSV, and influenza virus [69,70,71,72,73], and they mainly interfere with virus adsorption or internalization into host cells [72]. Recently, Buck *et al.* [7], demonstrated that carrageenans particularly ι-carrageenans can inhibit HPV infection three orders magnitude more potent than heparin, and mainly block the initial infection process of HPV. Carrageenan acts primarily by preventing the binding of HPV virions to cells and blocks HPV infection through a second, post attachment heparin sulfate-independent effect. Moreover, some of milk-based products, which contain carrageenan, were also reported to be able to block HPV infection *in vitro*, even when diluted million-fold [7]. In addition, carrageenan has been reported to inhibit genital transmission of HPV in female mouse model of cervicovaginal [74,75].

Based on these findings, carrageenan may be an alternative source of novel therapeutic candidate for HPV infection and some carrageenan-based sexual lubricant gels may be used to block the sexual transmission of HPV to some extend [7,74]. Moreover, there are some advantages of carrageenan over other classes of antiviral agents, such as low cytotoxicity, wide acceptability, and novel modes of action, which suggests that carrageenan merits further investigation as a promising anti-HPV agent in the future. However, most of the studies on anti-HPV effects of carrageenans have been observed *in vitro* or in mouse model systems, thus further studies with clinic trials are needed to determine whether carrageenan-based products are effective as topical microbicides against genital HPVs [7].

Furthermore, Buck *et al.* [7] reported that the sulfated polysaccharide agar derived from red algae could also effectively block HPV pseudovirion infection with the IC_50_ value of 0.27 µg/mL. In addition, carrageenan and its structurally related compounds such as dextran can also serve as adjuvants for enhancing peptide-based HPV vaccine potency [50]. Combined with the fact that iota carrageenans possess good anti-HPV activities *in vitro* and *in vivo*, we suppose that the sulfated galactose structure and the optimal sulfate content are very important for anti-HPV actions of carrageenans. In summary, the sulfated polysaccharides derived from red algae especially carrageenans merit further investigation as novel anti-HPV agents in the future.

### 3.3. Sulfated Polysaccharides from Brown Algae

It was reported that some sulfated polysaccharides from brown algae possess good antiviral and anti-tumor activities and some of them have been developed into novel antiviral agents [77,78,79,80,81]. Fucan is a term used to define a family of l-fucose-containing sulfated polysaccharides found in brown seaweed and the structures of these fucans vary among species and sometimes among different parts of the seaweed [82]. Some algal fucans exhibit important pharmacological activities such as anticoagulant [83], anti-inflammatory, antiviral [84], and antiproliferative [85]. It was found that the polysaccharide-rich extract from *Sargassum filipendula C. Agardh* showed significant anti-proliferative effect on HeLa cell (human uterine adenocarcinoma cell) proliferation [86]. In addition, a bioassay-guided fractionation of this extract led to the isolation of an antioxidant heterofucan denominated SF-1.5v, which exhibits good anti-proliferative activity against HeLa cells. However, the molecular mechanism underlying the heterofucan-induced anti-proliferative process remains unclear.

Moreover, Stevan *et al.* [87], reported that alginic acid isolated from the brown seaweed *Laminaria brasiliensis* (molar ratio M/G 1.2) and its M and G blocks (DP = 20) all could promote atypical mitoses in HeLa cells, besides the presence of acidophilic material in the cellular cytoplasm and occurrence of multinuclear cells present in the monolayer. In addition, the polysaccharide fraction SF isolated from the brown seaweed *Sargassum stenophyllum* containing mainly fucose could promote much accentuated morphologic modifications in HeLa cells at low concentrations (2.5 μg/mL) [87]. SF could cause significant alterations in the cellular morphology and reduction of cell growth in Hela cells in a dose dependent manner, which suggested this compound derived from brown algae could be used for treatment of HPV-related genital cancer in women in the future.

Furthermore, it was reported that fucoidan produced from brown algae could also inhibit HPV pesudovirus infection *in vitro* with the IC_50_ value of 1.1 µg/mL [7]. Although the anti-HPV effects of fucoidan are not as good as carrageenan (IC_50_ < 0.1 µg/mL), it also merits further investigation as novel anti-HPV or anti-cervical cancer candidate in the future. Taken together, brown algae-derived bioactive compounds, in particular the alginate polysaccharides and fucans, have good antiviral or anti-tumor activities, thus the sulfated polysaccharides from brown algae have the potential to become new resources for the development of anti-HPV and related cancer agents.

### 3.4. Agents from Marine Microbes

Marine-derived fungi have proven to be a promising source of bioactive metabolites and a growing number of marine fungi have been reported to produce bioactive secondary metabolites [88,89]. *Aspergillus* species are filamentous saprophytic fungi that can be found in almost all aerobic environments, which possessed antitumor, anti-inflammatory, antiviral and antibacterial activity [90]. Gliotoxin, one of the secondary metabolites produced by a number of *Aspergillus*, *Gliocladium* and *Penicillium* species, is a tricyclic alkaloid [91,92,93]. Gliotoxin effectively reduced the proliferation of HPV18 transformed Hela cells and could induce apoptotic cell death in association with the loss of mitochondrial membrane potential (MMP), and activation of Bax, caspase-3, caspase-8 and caspase-9, as well as suppression of Bcl-2 [94]. In a word, gliotoxin isolated from marine fungus *Aspergillus* sp. may induce apoptosis in HPV related cancer cells via the mitochondrial pathway followed by downstream events leading to apoptotic mode of cell death [94].

**Figure 3:**
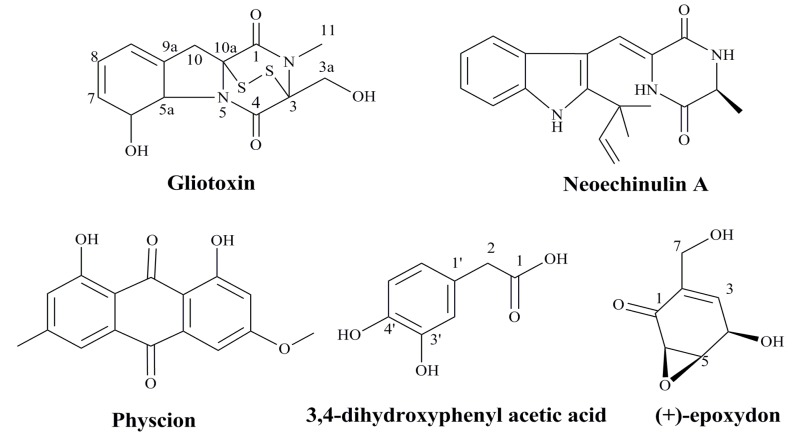
The structures of anti-HPV related cancer agents from marine microbes [94,95,96,97].

Marine microbes are valuable sources of structurally diverse bioactive compounds with anticancer activity. It was reported that a prenylated indole alkaloid, neoechinulin A, could be isolated from the culture broth extract of a marine-derived fungus, *Microsporum* sp. [95]. Neoechinulin A had good cytotoxic effect on human cervical carcinoma HeLa cells and it could induce cell apoptosis through down-regulating of Bcl-2 expression, up-regulating of Bax expression, and activating the caspase-3 pathway. Thus, neoechinulin A from marine-derived fungus merits further investigation as a potential candidate in the field of anticancer drug discovery against human cervical cancer [95]. In summary, some agents from marine microbes especially the bioactive metabolites have good inhibitory effects on the proliferation of HPV related cancer cells (Figure 3), which suggest these natural compounds can be developed into novel anti-HPV related cancer agents in the future.

### 3.5. Bioactive Compounds from Marine Animals

Marine life is a treasure trove of natural medicine, and the polysaccharides isolated from a variety of marine animals have been shown to possess good pharmacological activities such as antitumor, anti-inflammatory, and antiviral activity. Marine japonicus polysaccharide isolated from *Stichopus Japonicus* is a class of natural polysaccharides, which is rich in marine coelenterata. Some studies reported that marine japonicus polysaccharide SJAMP could significantly inhibit the proliferation of human cervical carcinoma Hela cell *in vitro* [98]. SJAMP could reduce the abnormal expression of proliferating cell nuclear antigen (PCNA) and cell cycle inhibitor protein Mdm2 protein, and induce cell cycle arrest in G1 phase, thus plays a role in the inhibition of HeLa cell proliferation. In a word, marine japonicus polysaccharide can inhibit HPV related cancer *in vitro* through interfering with the cell cycle of cervical cancer cell [98].

Moreover, Clam (*Meretrix meretrix Linnaeus*) is one of the main beach cultured shellfishes in China. Clams possess many biological activities, such as anti-tumor, antiviral, and antioxidant effects [99]. There are a variety of active natural products in Clams, such as proteins, peptides, polysaccharides, nucleic acids and sterols, and some of them can significantly inhibit tumor growth *in vitro* and *in vivo*. Zhang *et al.* [100] reported that a low molecular weight polypeptide Mer2 isolated from the body of Clam could effectively inhibit the growth of Hela cells in a dose-and time-dependent manner. Mer2 could induce obvious cell morphology change and the apoptosis phenomenon, but without a significant cell cycle arrest, which suggest that Mer2 probably inhibits the proliferation of cervical cancer cells by induction of cell apoptosis [100].

Furthermore, chitosan, a partially deacetylated polymer of *N*-acetylglucosamine, is produced by deacetylation of chitin derived from the shells of crabs and shrimps, and has been reported to have good pharmacological properties such as antiviral activities [65]. Recently, some researchers reported that a chitosan cervical antimicrobial film which contains mainly chitosan and gelatin possessed good inhibitory effects on chronic cervicitis combined HPV infection, and the HPV negative conversion rates in chitosan treated group were superior to that in interferon α-2b gel treated group after three courses of treatment [101,102]. Moreover, the diethylaminoethyl chitosan was found to be able to induce apoptosis in human cervix cancer Hela cells via up-regulation of caspases, p53, and Bax expression, and down-regulation of Bcl-2 expression [103]. In summary, some bioactive compounds from marine animals such as polysaccharides and polypeptides possess good inhibitory effects on HPV and its related cancer, which suggest these natural compounds merit further investigation as novel anti-HPV and related cancer agents in the future. In order to summarize the data available in the literature, marine derived anti-HPV and related cancer agents described in this paper were all shown in Table 1.

**Table 1:** Anti-HPV and related cancer agents from marine resources.

Marine Organisms	Specific Compounds	Mechanisms of Action	References
Red Algae	λ-carrageenan	Blocking HPV infection	[7,104]
	κ-carrageenan	Blocking HPV infection	[7,104]
	ι-carrageenan	Blocking HPV infection	[7,74,104]
	Agar	Blocking HPV infection	[7]
Brown Algae	Alginic acid	Inhibiting HPV and cancer cell proliferation	[7,87]
	Fucoidan	Blocking HPV infection	[7]
Marine Fungus	Gliotoxin	Inducing apoptosis in cancer cells	[94]
	Neoechinulin A	Inducing apoptosis in cancer cells	[95]
	Physcion	Inducing apoptosis in cancer cells	[96]
	(+)-epoxydon	Inducing apoptosis in cancer cells	[97]
Echinoderm	japonicus polysaccharide	Inducing apoptosis in cancer cells	[98]
Shellfish	Clam polypeptide	Inducing apoptosis in cancer cells	[100]
Crustacean	Chitosan	Inhibiting HPV and cancer cell proliferation	[101,102,103]

## 4. Prospects of Marine Derived Anti-HPV and Related Cancer Agents

HPV-caused cancer is a major health problem worldwide, especially in developing countries. The discovery of medicinal agents specifically capable of inhibiting HPV and related cancer is urgently required to prevent HPV infection. Marine derived bioactive compounds especially the heparinoid polysaccharides have similar pharmacological activities to the natural heparin, which can effectively block the entry process of HPV. Carrageenan-containing sexual lubricant gels were reported to be able to inhibit the infectivity of HPV16 pseudovirus *in vitro*, which suggests use of such carrageenan based gels may be able to block the sexual transmission of HPV [7]. Moreover, the anti-HPV related cancer agents produced by natural compounds often can not only target against the viral oncogenes but also inhibit the dysregulated gene expression of the host cells [14]. So natural products derived from marine organisms are excellent sources for the effective discovery of anti-HPV and related cancer agents.

However, the possibility that some genital HPVs might exhibit natural resistance to inhibition by carrageenan or other marine derived natural compounds would be an important factor to consider in the design of clinical efficacy trials [7]. In addition, despite having good anti-HPV activities, marine polysaccharides are structurally diverse and heterogeneous, which makes studies of their structures challenging, and may also have hindered their development as therapeutic agents to date [105]. Moreover, most of studies on anti-HPV activity of marine-derived HPV inhibitors have been observed *in vitro* or in mouse model systems until now. Therefore, further studies are needed to explore their activities in clinic in the future.

## 5. Conclusions

In summary, the anti-HPV and related cancer agents derived from marine organisms have the potential to treat both unapparent HPV infection as well as visible clinical diseases. Besides that, marine natural products especially polysaccharides and polypeptides have many advantages, such as relatively low cytotoxicity, low production costs, and wide acceptability, which suggest these compounds merit further investigation as novel drug candidates to reduce or regulate HPV infection related cancer [106,107]. Furthermore, the underlying molecular mechanisms of antiviral actions of marine-derived HPV inhibitors need to be elucidated clearly by intensive studies in the future [108].
